# LncRNAs: the bridge linking RNA and colorectal cancer

**DOI:** 10.18632/oncotarget.13573

**Published:** 2016-11-24

**Authors:** Yanfei Yang, Linjie Zhao, Lingzi Lei, Wayne Bond Lau, Bonnie Lau, Qilian Yang, Xiaobing Le, Huiliang Yang, Chenlu Wang, Zhongyue Luo, Yu Xuan, Yi Chen, Xiangbing Deng, Lian Xu, Min Feng, Tao Yi, Xia Zhao, Shengtao Zhou

**Affiliations:** ^1^ Department of Obstetrics and Gynecology, Key Laboratory of Birth Defects and Related Diseases of Women and Children of Ministry of Education, West China Second Hospital and State Key Laboratory of Biotherapy/Collaborative Innovation Center, West China Hospital, Sichuan University, Chengdu, P. R. China; ^2^ Department of Emergency Medicine, Thomas Jefferson University Hospital, U.S.A; ^3^ Department of Emergency Medicine, Kaiser Permanente Santa Clara Medical Center, Affiliate of Stanford University, U.S.A; ^4^ Department of Orthopedics, West China Hospital, Sichuan University, Chengdu, P. R. China; ^5^ College of Life Sciences, Sichuan University, Chengdu, P. R. China; ^6^ Department of Gastrointestinal Surgery, West China Hospital, Sichuan University, Chengdu, P.R. China; ^7^ Department of Pathology, West China Second Hospital, Sichuan University, Chengdu, P. R. China

**Keywords:** lncRNAs, colorectal cancer, proliferation, angiogenesis, metastasis

## Abstract

Long noncoding RNAs (lncRNAs) are transcribed by genomic regions (exceeding 200 nucleotides in length) that do not encode proteins. While the exquisite regulation of lncRNA transcription can provide signals of malignant transformation, lncRNAs control pleiotropic cancer phenotypes through interactions with other cellular molecules including DNA, protein, and RNA. Recent studies have demonstrated that dysregulation of lncRNAs is influential in proliferation, angiogenesis, metastasis, invasion, apoptosis, stemness, and genome instability in colorectal cancer (CRC), with consequent clinical implications. In this review, we explicate the roles of different lncRNAs in CRC, and the potential implications for their clinical application.

## INTRODUCTION

Colorectal cancer is a frequently diagnosed and fatal malignancy worldwide, with nearly 1.4 million newly diagnosed cases in 2012 [1]. The conventional chemotherapeutic strategies for CRC involve the use of highly toxic drugs, which cause undesirable side-effects [2]. It is therefore important to identify both novel biomarkers and therapies for early diagnosis and improved treatment.

**Figure 1:**
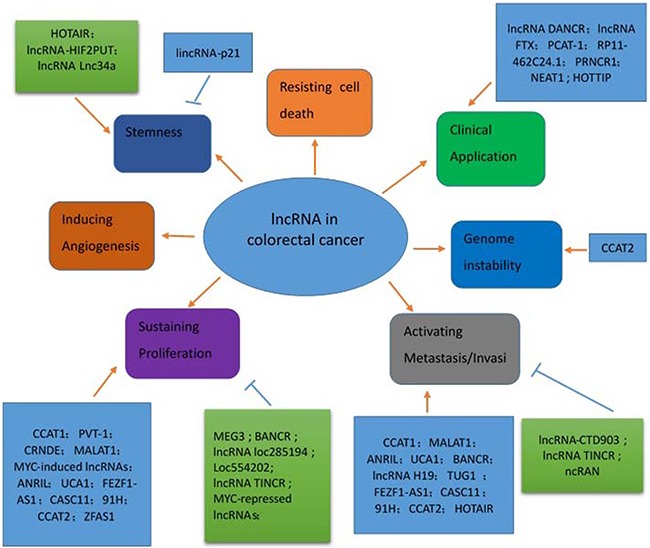
The functions of lncRNA in colorectal cancer pathogenesis LncRNAs have multiple functions in the context of colorectal cancer, including increasing proliferation, angiogenesis, metastasis, invasion, apoptosis, stemness, and genomic instability. Red arrows indicate promoted signaling pathways. Blue arrows indicate inhibited signaling pathways. Some LncRNAs have multiple functions (for example, CCAT2 promotes proliferation, metastasis, and genomic instability). *Abbreviations: CCAT2: colon cancer-associated transcript 2; CCAT1: LncRNA colon cancer-associated transcript 1; BANCR: BRAF-activated lncRNA; TUG1: Taurine up-regulated gene 1; FEZF1-AS1: long noncoding RNA FEZF1 antisense RNA1; CASC11: cancer susceptibility candidate 11; CRNDE: Colorectal Neoplasia Differentially Expressed; UCA1:urothelial carcinoma-associated 1 ;MEG3: maternally expressed gene 3; MALAT1: metastasis associated lung adenocarcinoma transcript 1; ANRIL: Antisense noncoding RNA in the INK4 locus; HOTAIR: Hox transcript antisense intergenic RNA; HOTTIP: ‘HOXA transcript at the distal tip’*

Recently, the genomics has identified unexpected non-protein coding regions of the genome. The list of long noncoding RNAs (lncRNAs), functionally defined as transcripts >200 nt in length with no protein-coding potential, continues to grow. Researchers have discovered many of these lncRNAs are uniquely expressed in differentiated tissues or specific cancer types. It is now established that lncRNAs are exquisitely controlled and are restricted to specific cell types to a greater degree than mRNA [3]. They frequently have evolutionarily conserved function, secondary structure, and regions of microhomology, despite minimal overall sequence similarity [4]. However, the functions of the vast majority of these transcripts remain unknown. Evidence supports the important role lncRNAs perform in CRC (Figure 1), via incompletely understood mechanisms. In this review, we comprehensively detail the functional roles of lncRNAs in maintaining the hallmarks of malignancy in colorectal cancer, and their potential clinical applications in the treatment of CRC (Table 1).

**Table 1:** A summary of lncRNAs involved in colorectal cancer development and progression

LncRNA	In colorectal cancer, its expressions is:	Functions	Reference
Colon Cancer Associated Transcript 1 (CCAT1)	Upregulated	Promotes cellular proliferation, migration, and invasion.	[6]
Plasmacytoma variant translocation 1(PVT-1)	Upregulated	Promotes proliferation and invasion. Knockdown of PVT-1 promotes apoptosis in colorectal cancer cell lines by activating the TGF-β signaling pathway.	[7]
Colorectal Neoplasia Differentially Expressed (CRNDE)	Upregulated	Promotes colon cancer cell proliferation.	[8, 9, 10]
Metastasis Associated Lung Adenocarcinoma Transcript 1 (MALAT1)	Upregulated	Promotes cellular proliferation, migration, and invasion via 1) binding to SFPQ and releasing oncogene PTBP2 from SFPQ/PTBP2 complex 2) increasing expression of AKAP-9 via promoting SRPK1-catalyzed SRSF1 phosphorylation in colorectal cancer cells.	[11, 12, 13]
ZNFX1 antisense RNA 1 (ZFAS1)	Upregulated	Silencing of ZFAS1 decreases CRC cell proliferation via G1-arrest of the cell cycle and decreasing CRC tumorigenicity. ZFAS1 acts as an oncogene in CRC via 1) destabilization of p53; 2) interaction with CDK1/cyclin B1 complex leading to cell cycle progression and suppression of apoptosis.	[14, 15, 16, 17]
MYC-induced lncRNAs (MYCLo-1, MYCLo-2, MYCLo-3)	Upregulated	Promotes MYC-modulated cell proliferation. MYCLo-1/−2 promote G1/S transition. MYCLo-3 decreases cellular time spent in the S and G2 phases.	[18]
MYC-repressed lncRNAs (MYCLo-4, MYCLo-5, MYCLo-6)	Downregulated	Inhibits MYC-enhanced cell proliferation. MYCLo-4/−6 increase G2 arrest. MYCLo-5 decreases cellular populations in the S phase.	[19]
Antisense noncoding RNA in the INK4 locus (ANRIL)	Upregulated	Promotes proliferation in a p15/p16-pRB pathway-independent manner, and promotes cell invasion and migration.	[20, 61]
urothelial carcinoma-associated 1 (UCA1)	Upregulated	Activates proliferation, suppresses apoptosis and cell cycle progression of CRC cells. UCA1 induces CRC migration and invasion and predicts poor prognosis.	[21, 22, 23]
BRAF-activated lncRNA (BANCR)	Downregulated	Inhibits the proliferation in part through upregulation of p21, induces the epithelial-mesenchymal transition (EMT) through an MEK/extracellular signal-regulated kinase-dependent mechanism.	[24, 33]
Maternally expressed gene 3 (MEG3)	Downregulated	Inhibits CRC cell proliferation and is an independent predictor for overall survival.	[25]
LncRNA loc285194	Downregulated	Suppresses tumor cell growth due to specific suppression of miR-211. Low expression of LOC285194 is associated with larger tumor size, higher tumor stage, more distant metastasis and poorer disease free survival.	[26, 27, 28]
Loc554202	Downregulated	Suppresses the cell proliferation, induces apoptosis, partly through activating specific caspase cleavage cascades, and inhibits CRC tumorigenesis.	[32]
LncRNA H19	Upregulated	Promotes EMT in CRC.	[34]
Hox transcript antisense intergenic RNA (HOTAIR)	Upregulated	Indicates poorer prognosis, promotes migration and invasion, enhances CSC properties, promotes cellular proliferation, decreases the expression of E-cadherin and increases expression of vimentin and MMP9.	[36, 49]
HOTTIP(‘HOXA transcript at the distal tip’ )	Upregulated	Predicts unfavorable prognosis for CRC patients.	[37]
Taurine up-regulated gene 1 (TUG1)	Upregulated	Increases the invasive and metastatic ability of CRC cells through activating EMT process and TUG1 overexpression indicates poor survival rates and a higher risk for cancer metastasis.	[38]
FEZF1 antisense RNA1 (FEZF1-AS1)	Upregulated	Promotes migration and proliferation through activating the G1-S checkpoint	[39]
Cancer susceptibility candidate 11 (CASC11)	Upregulated	Promotes CRC cell proliferation and metastasis by activation of WNT/β-catenin signaling.	[40]
91H	Upregulated	Promotes the proliferation, migration, and invasiveness of CRC cells.	[41]
LncRNA-CTD903	Downregulated	Predicts favorable prognosis in CRC patients and suppresses invasion and migration through repressing Wnt/β-catenin signaling.	[42]
LncRNA TINCR	Downregulated	Suppresses CRC proliferation and metastasis by accelerating the cleavage of EpCAM and releases EpICD via activating WNT/β- catenin pathway.	[43]
ncRAN	Downregulated	Inhibits in vitro migration and invasion of CRC cells and predicts CRC patient outcome.	[44]
Colon cancer-associated transcript 2 (CCAT2)	Upregulated	Promotes tumor growth, metastasis, and chromosomal instability. It functions as a WNT downstream target.	[46]
LncRNA-HIF2PUT	Upregulated	Promotes the HIF-2α expression and the CSC properties.	[48]
Lnc34a(locus mainly in the nucleus)	Upregulated	Enhances CSC self-renewal and tumorigenesis and suppresses miR-34a expression.	[50]
LincRNA-p21	Downregulated	Attenuates the viability, self-renewal, and glycolysis of CSCs.	[51]
LncRNA DANCR	Upregulated	Serves as a potential prognosis predictor for CRC prognosis, associates with TNM stage, histologic grade, and lymph node metastasis, and predicts shorter overall survival and disease-free survival time.	[52]
LncRNA FTX	Upregulated	Serves as an independent prognostic factor for CRC patients and is associated with differentiation grade, lymph vascular invasion, and clinical stage; indicates poorer overall survival.	[53]
Prostate cancer associated transcript 1(PCAT-1)	Upregulated	Functions as an independent prognostic factor for CRC patient outcome and implicates poorer overall survival.	[54, 55]
RP11-462C24.1	Downregulated	Serves as a prognosis indicator for CRC patients. Its down-regulation indicates increased distant metastasis and a poor disease-free survival.	[56]
PRNCR1	Upregulated	Serves as a sensitive diagnostic biomarker of CRC.	[57]
NEAT1	Upregulated	Functions as s diagnostic and prognostic biomarker of overall survival in CRC, associates with tumor differentiation, invasion, metastasis and TNM stage and predicts shorter disease-free survival time and overall survival time.	[58, 59]

## SUSTAINING PROLIFERATION

In normal tissue, the production and release of cellular growth signals is tightly controlled, ensuring homeostasis and balance in the cellular population. Dysregulation of such signaling leads to malignancy. Uncontrolled gene transcription with resultant protein production may increase cellular proliferation, but excess proliferative signaling may in turn trigger cellular senescence. Cancer cells may also sustain proliferation by evading growth suppressors. For instance, the RB protein is pivotal in negative control of the cell cycle and tumor progression. TP53 proteins control cellular division and prevent uncontrolled growth-and-division by activating apoptosis and preventing cell-cycle progression [5]. In recent years, the role of lncRNAs in sustaining proliferation of CRC cells has garnered great interest (Table 2).

**Table 2:** LncRNAs involved in sustained proliferation of CRC

LncRNA	In colorectal cancer, its expressions is:	Functions	Reference
LncRNA	Expression	Functions	Reference
CCAT1	Upregulated	Promotes colon cancer cell proliferation.	[6]
PVT-1	Upregulated	Promote proliferation and invasion; Knockdown of PVT-1 promotes apoptosis in colorectal cancer cell lines by activating the TGF-β signalling pathway.	[7]
CRNDE	Upregulated	Promotes colon cancer cell proliferation.	[8, 9, 10]
MALAT1	Upregulated	Promotes cellular proliferation, migration, and invasion via 1) binding to SFPQ and releasing oncogene PTBP2 from SFPQ/PTBP2 complex 2) increasing expression of AKAP-9 via promoting SRPK1-catalyzed SRSF1 phosphorylation in colorectal cancer cells.	[11, 12, 13]
ZFAS1	Upregulated	Silencing of ZFAS1 decreases CRC cell proliferation via G1-arrest of the cell cycle and decreasing CRC tumorigenicity. ZFAS1 acts as an oncogene in CRC via 1) destabilization of p53; 2) interaction with CDK1/cyclin B1 complex leading to cell cycle progression and suppression of apoptosis.	[14, 15, 16, 17]
MYC-induced lncRNAs (MYCLo-1, MYCLo-2, MYCLo-3)	Upregulated	Promotes MYC-modulated cell proliferation. MYCLo-1/−2 promote G1/S transition. MYCLo-3 decreases cellular time spent in the S and G2 phases	[18]
MYC-repressed lncRNAs (MYCLo-4, MYCLo-5, MYCLo-6)	Downregulated	Inhibits MYC-enhanced cell proliferation. MYCLo-4/−6 increase G2 arrest. MYCLo-5 decreases cellular populations in the S phase.	[19]
ANRIL	Upregulated	Promotes proliferation in a p15/p16-pRB pathway-independent manner.	[20]
UCA1	Upregulated	Activates proliferation, suppresses apoptosis and cell cycle progression of CRC cells.	[21, 22, 23]
BANCR	Downregulated	Inhibits the proliferation in part through upregulation of p21	[24]
Maternally expressed gene 3 (MEG3)	Downregulated	Inhibits CRC cell proliferation and serves as an independent predictor for overall survival.	[25]
LncRNA loc285194	Downregulated	Suppresses tumor cell growth due to specific suppression of miR-211. Low expression of LOC285194 shows larger tumor size, higher tumor stage, more distant metastasis and poorer disease free survival.	[26, 27, 28]
Loc554202	Downregulated	Inhibits the cell proliferation and induces apoptosis, partly through activating specific caspase cleavage cascades.	[32]
Long noncoding RNA FEZF1 antisense RNA1 (FEZF1-AS1)	Upregulated	Promotes proliferation through activating the G1-S checkpoint.	[39]

LncRNA colon cancer-associated transcript 1(CCAT1) is a recently discovered 2628 nucleotide-lncRNA, located in the vicinity of c-Myc. He et al discovered that c-Myc could promote CCAT1 transcription by directly binding to its promoter region, and enhanced CCAT1 expression in CRC cells cells increased cell proliferation and invasion [6]. Moreover, Takahashi et al reported lncRNA Plasmacytoma variant translocation 1 (PVT-1) expression is increased by amplification of 8q24 copy-number. CRC cells transfected with PVT-1 specific siRNA exhibited significant proliferative and invasive capability loss [7], supporting PVT-1 as a potential carcinogenic lncRNA in CRC. Another oncogenic lncRNA regulating CRC proliferation is Colorectal Neoplasia Differentially Expressed (CRNDE) [8], which is located on chromosome 16 of the human genome. It shares a bi-directional promoter with iroquois homeobox 5 (IRX5), which is adjacent at the opposite strand. The expression of CRNDE is tissue-specific, and follows a temporal pattern. Rarely is CRNDE observed in adult colorectal mucosa, liver, and white blood cells, and increased CRNDE expression occurs in the testis, breast, and skin [9]. Additionally, CRNDE expression is significantly increased in several neoplastic diseases, including colorectal cancer. There are two existing CRNDE transcripts: cytoplasmic (fully spliced and free of intron sequences) and nuclear (the intron sequences) transcripts. Insulin, IGF1, and IGF2 do not increase or decrease cytoplasmic CRNDE transcripts, but decrease nuclear CRNDE transcripts *via* both the PI3K/Akt/mTOR and Raf/MAPK pathways. Knockdown of gVC-In4, a highly-conserved region of CRNDE, affects insulin signaling responses, as well as many genes associated with glucose/lipid metabolism. Several other insulin-regulated genes are affected by gVC-In4 knockdown as well. GLUT4, an insulin-regulated glucose transporter, translocates to the plasma membrane, allowing intracellular glucose transport. CRNDE increases GLUT4 and MLXIPL transcription. gVC-In4 knockdown decreases glucose intake, thereby causing less lactate secretion. The increased expression of CRNDE nuclear transcripts promotes glucose metabolism, lactate secretion, and lipid synthesis in CRC cells. CRNDE nuclear transcripts influence upstream insulin/IGF signaling pathways. Thus CRNDE nuclear transcripts, which are inhibited by insulin, IGF1, and IGF2 via PI3K/Akt/mTOR and Raf/MAPK pathways, promote central metabolism [10]. Another well known lncRNA overexpressed in CRC is the human metastasis associated lung adenocarcinoma transcript 1 (MALAT1) [11]. Xu et al first reported that one fragment (6918 nt-8441 nt, located at the 3’ end) of MALAT-1 is influential in the biological processes of cell proliferation, migration, and invasion [11]. Several mechanisms connect MALAT1 to CRC cellular proliferation. It has been reported that MALAT1 overexpression enhances cell proliferation and migration in vitro, and promotes tumor growth and metastasis in nude mice, due to tumor suppressor gene SFPQ and proto-oncogene PTBP2. MALAT1 binds SFPQ, releasing PTBP2 from the SFPQ/PTBP2 complex. In turn, increased levels of SFPQ-detached PTBP2 promote cell proliferation and migration. In this sense, SFPQ mediates the regulatory effects of MALAT1 [12]. Furthermore, MALAT1 interacts with both SRPK1 and SRSF1. MALAT1 increases AKAP-9 expression by promoting SRPK1-catalyzed SRSF1 phosphorylation. After MALAT1 knockdown, overexpression of SRPK1 restored SRSF1 phosphorylation and AKAP-9 expression, promoting cell proliferation, invasion, and migration in vitro. Conversely, SRPK1 knockdown after overexpression of MALAT1 in a CRC cell line reduced SRSF1 phosphorylation and AKAP-9 expression, and inhibited cell proliferation, invasion, and migration in vitro. These findings suggest MALAT1 increases AKAP-9 expression by promoting SRPK1-catalyzed SRSF1 phosphorylation in CRC cells [13].

Thorenoor et al recently profiled expression of disease-associated lncRNAs in CRC tumor tissues and identified ZFAS1 (zinc finger antisense 1), previously known to be a tumor suppressor gene in human breast cancer [14, 15] and oncogene in hepatocellular carcinoma [16], to be overexpressed in CRC tissue. ZFAS1 silencing reduced CRC cell proliferation through G1-arrest of cell cycle, and also tumorigenicity of CRC cells. Via RIP (RNA immunoprecitation) analysis, the authors identified Cyclin-dependent kinase 1 (CDK1) as an interacting partner of ZFAS1. Through bioinformatics analysis, the authors found that ZFAS1 could sponge miR-590-3p, which targets CDK1. The authors further demonstrated ZFAS1 activates CDK1 and cyclin B1. When ZFAS1 is silenced, cyclin B1 is decreased. ZFAS1 silencing activates P53 and PARP cleavage, increasing CRC cell apoptosis. Taken together, ZFAS1 advances cell cycle progression and inhibits apoptosis, via activation of P53 and CDK1/cyclin B1 complex, thus critical for CRC cell viability, cell cycle distribution, apoptosis, and colony formation [17]. Kim recently identified MYCLos as a key molecule in CRC cell cycle regulation and tumorigenesis, by influencing MYC target genes such as CDKN1A (p21) and CDKN2B (p15) [18]. RNA binding proteins HuR and hnRNPK are involved in the function of MYCLos, by respectively interacting with MYCLo-1 and MYCLo-2. Knockdown of MYCLo-2, differentially expressed in CRC, inhibits cancer transformation and tumorigenesis [18]. The same research group later characterized another three MYC-repressed lncRNAs named MYCLo-4, -5, and -6. The MYC-repressed MYCLos were proved to suppress cell cycle progression, and therefore inhibiting cell proliferation. Through screening all cell cycle-related genes affected by MYC and MYC-repressed MYCLos, the MYC-repressed gene GADD45A has been identified to be a target gene of MYCLo-4 and MYCLo-6 [19].

ANRIL is a lncRNA transcribed from the INK4 locus, and encodes three tumor suppressor genes (p15, p16, and ARF). ANRIL represses p15 and p16, which positively regulate the pRB pathway, repressing senescence of normal human fibroblasts [20]. However, the role of ANRIL in cancer cell proliferation is not well understood. Naemura et al reported ANRIL increased proliferation of colorectal cancer HCT116 cells in two- and three-dimensional cultures. Silencing ANRIL by siRNA and retroviral-produced shRNA decreased HCT116 cell proliferation in both two- and three-dimensional cultures. HCT116 cells depleted of ANRIL were arrested in the S phase of the cell cycle. Notably, silencing ANRIL did not activate expression of the INK4 locus. These observations suggest ANRIL increases proliferation of HCT116 cells in two- and three-dimensional cultures via p15/p16-pRB [20].

The lncRNA urothelial carcinoma-associated 1 (UCA1) is significantly overexpressed in most tumor tissues and cancer cells. Han et al demonstrated UCA1 levels were markedly increased in CRC tissues and cells compared to control, and the level of UCA1 expression was positively correlated with tumor size, poor histological differentiation, and increased tumor depth. Increased UCA1 expression carried significantly poorer prognosis and overexpression of UCA1 increases proliferation, and decreases apoptosis and cell cycle progression of CRC cells [21]. Ni et al further demonstrated that increased UCA1 expression was positively correlated to lymph node metastasis, distant metastasis, and tumor stage. Survival analysis revealed correlation between increased UCA1 expression and poor prognosis. Moreover, multivariate analysis identified UCA1 overexpression as an independent predictor for CRC. UCA1 knockdown significantly decreased CRC proliferation and metastasis. Flow cytometry assays demonstrated UCA1 silencing induced G0/G1 growth arrest and apoptosis of CRC cells. To further investigate the regulatory mechanisms of UCA1, the authors identified that Ets-2 bound to the UCA1 core promoter using luciferase assays [22]. This observation suggested that UCA1 might be an important prognostic predictor in CRC and might be considered as a potential target for CRC diagnosis and gene therapy. More recently, Bian et al reported UCA1 sponged endogenous miR-204-5p, thereby inhibiting its activity in CRC, and identified CREB1 as a new target of miR-204-5p. The protein levels of CREB1 were significantly increased in CRCs, positively correlated with UCA1 expression, and negatively associated with survival. Taken together, Bian et al's work demonstrated existence of a UCA1-miR-204-5p-CREB1/BCL2/RAB22A regulatory network in CRC, supporting UCA1 and CREB1 as potential new oncogenes and prognostic factors for CRC [23].

LncRNAs may serve as tumor suppressors inhibiting CRC cellular proliferation. BRAF-activated non-coding RNA (BANCR) was first discovered during an RNA-seq screen for transcripts influenced by the expression of the oncogene BRAFV600E. Shi et al demonstrated significantly decreased expression of BANCR in three colorectal cancer cell lines. Suppression of BANCR increases proliferation of colorectal cancer cells, partly via downregulation of p21 [24]. Maternally expressed gene 3 (MEG3) is another lncRNA that functions as a CRC proliferative suppressor. Yin et al reported decreased MEG3 levels correlated positively with low histological grade, increased tumor invasion, and advanced tumor node metastatic (TNM) stage CRC disease. Multivariate analyses revealed that MEG3 expression served as an independent predictor for overall survival. Further experiments demonstrated MEG3 overexpression significantly inhibited CRC cell proliferation both in vitro and in vivo [25].

Two lncRNAs have recently been identified as CRC proliferation inhibitors. Pasic et al first reported in 2010 lncRNA loc285194 to be within a tumor suppressor unit in osteosarcoma, and suppressed tumor cell growth [26]. Another research group demonstrated LOC285194 to be decreased in tumor tissues and colorectal cancer cell lines compared to normal intestinal mucous cell lines. Additionally, low levels of LOC285194 were correlated with larger tumor size, increased tumor stage, more distant metastasis, and decreased disease-free survival [27]. Liu et al demonstrated loc285194 is a p53 transcription target, and ectopic expression of loc285194 inhibits tumor cell growth both in vitro and in vivo. Through deletion analysis, Liu et al identified an active region responsible for tumor cell growth inhibition within exon 4, which harbors two miR-211 binding sites. This loc285194-mediated growth inhibition is partially due to specific suppression of miR-211 [28]. The second newly discovered CRC inhibitor lncRNA is loc554202. Loc554202 is a 2166-bp transcript on human chromosome 9p21.3, previously shown to be dysregulated in breast and lung cancer [29–31]. Ding et al reported expression of Loc554202 was significantly inhibited in CRC cell lines compared to normal human intestinal epithelial cell lines. Decreased loc554202 expression was associated with advanced pathologic stage and a larger tumor size. Moreover, loc554202 expression decreased cellular proliferation, induced apoptosis in vitro, and hindered tumorigenesis in vivo, partly via specific caspase cleavage cascade activation [32].

## ACTIVATING METASTASIS/INVASION PROGRAM

**Figure 2:**
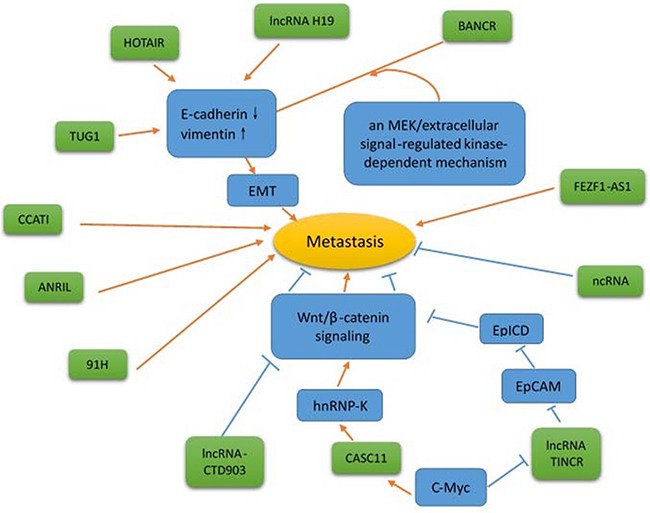
The regulation of metastasis by lncRNA Red arrows indicate promoted signaling pathways; blue arrows indicate inhibited signaling pathways. Several lncRNAs (such as CCAT1, ANRIL, 91H, FEZF1-AS1, and ncRNA) have unknown mechanisms.

Although primary CRC can be treated by surgical resection, patient survival deteriorates once metastasis to vital organs (such as the liver or lungs) has occurred. Primary CRC develops progressively through accumulation of genetic mutations (e.g., APC, KRAS, p53, SMAD4, and PTEN) and epigenetic silencing of tumor suppressor genes. Mounting evidence suggests lncRNAs influence the invasive and metastatic potential of CRC (Figure 2 and Table 3). For instance, Guo et al. determined BANCR is overexpressed in CRC tissues, correlating with increased lymph node metastasis and tumor stage. While ectopic BANCR expression increased migration of human CRC Caco-2 cells, BANCR knockdown inhibited in vitro HCT116 cell migration [33], via induction of the epithelial-mesenchymal transition (EMT) through a MEK/extracellular signal-regulated kinase-dependent mechanism. These results suggest BANCR is essential for CRC metastasis, with great potential therapeutic value against CRC progression.

**Table 3:** LncRNAs participate in metastasis/invasion-promoting programs of CRC

LncRNA	In colorectal cancer, its expressions is:	Functions	Reference
CCAT1	Upregulated	Promotes cell migration and invasion.	[6]
BANCR	Upregulated	Induces EMT program through an MEK/extracellular signal-regulated kinase-dependent mechanism.	[33]
LncRNA H19	Upregulated	Promotes EMT in CRC.	[34]
HOTAIR	Upregulated	Promotes migration and invasion, decreases expression of E-cadherin, increases expression of vimentin and MMP9 and associates with poorer prognosis.	[36]
TUG1	Upregulated	Increases the invasive and metastatic ability of CRC cells through activating EMT process	[38]
FEZF1-AS1	Upregulated	Promotes migration.	[39]
CASC11	Upregulated	Promotes CRC cell metastasis, dependent on activation of WNT/β-catenin signaling.	[40]
91H	Upregulated	Promotes proliferation, migration, and invasiveness of CRC cells.	[41]
LncRNA-CTD903	Downregulated	Suppresses invasion and migration through repressing Wnt/β-catenin signaling.	[42]
LncRNA TINCR	Downregulated	Suppresses CRC proliferation and metastasis by accelerating the cleavage of EpCAM and releasing EpICD via activating WNT/β- catenin pathway.	[43]
ncRAN	Downregulated	Inhibits migration and invasion of CRC cells.	[44]
ANRIL	Upregulated	Promotes cell invasion and migration.	[61]

The lncRNA H19, a known oncogene in various cancer types, tumorigenesis and cancer progression. Liang et al recently characterized H19 as a novel regulator of epithelial-to-mesenchymal transition (EMT) in CRC. H19 is highly expressed in mesenchymal-like cancer cells and primary CRC tissues. Stable H19 expression promotes EMT progression and accelerates in vivo and in vitro tumor growth. Bioinformatics in combination with RIP analysis revealed H19 functions as a competing endogenous RNA (ceRNA) for miR-138 and miR-200a, antagonizing their function, and activating their endogenous targets Vimentin, ZEB1, and ZEB2, all key marker genes of mesenchymal cells. In summary, lncRNA H19 is involved in EMT program as a competing endogenous RNA, ultimately contributing to CRC progression [34].

HOTAIR (the Hox transcript antisense intergenic RNA), is a lncRNA expressed from the developmental HOXC locus located on chromosome 12q13.13. Kogo et al reported HOTAIR expression levels were higher in cancerous tissues than in noncancerous tissues, and increased HOTAIR expression correlated positively with the liver metastasis. Moreover, patients expressing increased HOTAIR levels had poor prognosis. In a subset of 32 CRC specimens, gene set enrichment analysis using cDNA array data revealed a close correlation between expression of HOTAIR and members of the PRC2 complex (SUZ12, EZH2, and H3K27me3) [35]. Wu et al more recently demonstrated HOTAIR was associated with epithelial-mesenchymal transition in CRC, reporting HOTAIR depletion increased the expression of E-cadherin while concomitantly decreasing expression of vimentin and MMP9 [36]. Upregulation of HOTAIR might be a critical element contributing to CRC metastatic progression. LncRNA taurine up-regulated gene 1 (TUG1) has been reported to be correlated with cancer progression. Sun et al specifically analyzed TUG1 in the context of CRC, and demonstrated TUG1 expression was significantly increased in CRC cell lines, suggesting TUG1 downregulation may be a negative prognostic factor for CRC patients. TUG1 may increase the invasive and metastatic ability of CRC cells, at least in part through influencing the EMT process [38].

LncRNA FEZF1 antisense RNA1 (FEZF1-AS1) was recently corroborated to be significantly oveexpressed in human primary CRC, and is associated with CRC metastasis and poor prognosis. Moreover, suppression of FEZF1-AS1 expression significantly inhibited CRC cell proliferation, migration and invasiveness, suppressed S-phase entry in vitro, and repressed tumor growth and metastasis in vivo. FEZF1-AS1 overexpression increased malignant aspects of CRC cells. Dysregulation of FEZF1-AS1 therefore participates in CRC tumorigenesis and progression [39]. Moreover, The overexpression of FEZF1-AS1 leads to significant cells reduction at G0/G1-phase and increase in S-phase. Conversely, knockdown of FEZF1-AS1 does just the opposite. FEZF1-AS1 induces CRC cell proliferation by activating the G1-S checkpoint [39].

Cancer susceptibility candidate 11 (CASC11) is overexpressed in CRC tissues and is positively associated with tumor size, serosal invasion, lymph metastasis, and tumor-node-metastasis (TNM) stage. CASC11 promotes CRC cell proliferation and metastasis in vitro and in vivo. CASC11 increases expression of heterogeneous ribonucleoprotein K (hnRNP-K) and β-catenin nuclear accumulation, activating WNT/β-catenin signaling in CRC cells [40]. Additionally, c-Myc directly binds the promoter regions of CASC11, increasing promoter histone acetylation, augmenting CASC11 expression. CASC11 has potential as a diagnostic biomarker, and may be an effective therapeutic against CRC. In addition, located on the H19/IGF2 locus (119.329 kbs long), 91H (H19 antisense RNA) is a lncRNA which is overexpressed in CRC tissue and cell lines. 91H overexpression is correlated with increased metastasis and poor prognosis. Multivariate analysis demonstrates 91H expression is an independent prognostic and metastatic indicator of CRC. Moreover, knockdown of 91H inhibited the proliferation, migration, and invasiveness of CRC cells [41].

Several lncRNAs that suppress metastasis were recently discovered. lncRNA-CTD903 is an independent predictor of favorable prognosis in CRC patients. After knockdown of CTD903 in CRC cell lines, CRC cells were noted to exhibit EMT-like appearance, decreased adherence ability, and increased invasion and migration characteristics. Inhibition of CTD903 increased Wnt/β-catenin activation, increased transcription factors Twist and Snail expression, increased mesenchymal marker Vimentin, and decreased epithelial marker ZO-1. LncRNA-CTD903 is therefore likely a suppressor of metastasis in CRC via Wnt/β-catenin signaling repression [42]. Another suppressor of CRC metastasis is TINCR, a 3.7 kb lncRNA. TINCR levels are negatively correlated with CRC progression, tumor growth, and metastasis (both in vivo and in vitro). During physiologic conditions, TINCR binds EpCAM. Loss of TINCR increases hydrolysis of EpCAM, increases release of EpICD, and activates the Wnt/β-catenin pathway. c-Myc decreases TINCR expression via repression of sp1-transcriptive activity, establishing a positive feedback loop controlling c-Myc and TINCR expression. Loss of TINCR expression promotes proliferation and metastasis in CRC, and is therefore a potential cancer suppressor gene [43]. ncRNA (non-coding RNA expressed in aggressive neuroblastoma) has previously been demonstrated to be overexpressed and associated with poor prognosis in human neuroblastoma, as well as bladder cancer. However, ncRAN is significantly decreased in CRC tumor tissue and CRC cell lines. Moreover, expression of ncRAN is inhibited in poorly differentiated or undifferentiated CRC tumors, and in CRC tumors with liver metastases. Multivariate analysis demonstrates decreased ncRAN expression is an independent predictor of overall survival. Taken together, these data suggest ncRAN may be a biomarker for early CRC metastasis, a prognostic indicator, and a therapeutic target against CRC [44].

## GENOME INSTABILITY

All organisms require faithful propagation of genetic material and transmission into progeny cells, and avoidance of mutation propagation that may lead to genomic instability and aberrant cellular activity. Various DNA insults may occur by extrinsic factors such as ultraviolet (UV) radiation, ionizing radiation (IR), and numerous genotoxic chemicals [45]. In cancer cells, the rates of mutation increase due to breakdown of the genomic maintenance machinery. Despite cancer cell adaptation to this increased mutation rate, there is inevitably genomic instability within tumors themselves. Unfortunately, few investigations have identified potential lncRNAs involved in this process in CRC (Table 4). Ling et al reported that colon cancer-associated transcript 2 (CCAT2), a novel lncRNA encompassing the rs6983267 SNP, was overexpressed in microsatellite-stable colorectal cancer and promoted tumor growth, metastasis, and chromosomal instability. MYC, miR-17-5p, and miR-20a are upregulated by CCAT2 via TCF7L2-mediated transcriptional regulation. The interaction between CCAT2 and TCF7L2 enhances WNT signaling. As CCAT2 is a WNT downstream target, a feedback loop likely exists. SNP status affects CCAT2 expression, and allele G increases CCAT2 transcript production. CCAT2 activates WNT signaling by enhancing TCF7L2 transcriptional activity to increase MYC expression. CCAT2 transcription increases WNT target gene expression by binding TCF7L2 and modulating its transcriptional activity. CCAT2 likely promotes chromosomal instability and tumor growth by activating WNT signaling and increasing MYC expression via activation of CDC25A, miR-17-5p, and miR-20a) [46].

**Table 4:** Genome instability-related lncRNAs

LncRNA	In colorectal cancer, its expressions is:	Functions	Reference
CCAT2	Upregulated	Increases chromosomal instability.	[46]

## STEMNESS

CRC has been recently identified to encompass a subset of cells with stem/progenitor cell features known as cancer stem cells (CSCs), which could lead to advanced tumors and a poor prognosis [47]. Because CSCs contribute to tumor-initiating potential, invasion, metastasis, resistance to traditional therapies, and eventual relapse, the CSC model explains clinical events previously a mystery, including therapy resistance, minimal residual disease, and tumor recurrence. However, the molecular hallmarks of CSCs in colorectal cancer remain unelucidated. While lncRNAs have been identified to be intimiately involved with various malignancies, their role in stemness maintenance in CRC remain the subject of intense study (Table 5).

**Table 5:** LncRNAs associated with stemness mantenance in CRC

lncRNA	In colorectal cancer, its expressions is:	Functions	Reference
LncRNA-HIF2PUT	Upregulated	Enhances the HIF-2α expression and promotes CSC properties.	[48]
LncRNA HOTAIR	Upregulated	Promotes CSC properties, increases cellular proliferation, migration, invasion, colony-forming.	[49]
Lnc34a(locus mainly in the nucleus)	Upregulated	Enhances CSC self-renewal and tumorigenesis and suppresses miR-34a expression.	[50]
LincRNA-p21	Downregulated	Attenuates the viability, self-renewal, and glycolysis of CSCs.	[51]

The lncRNA “lncRNA-HIF2PUT” is a promoter upstream transcript of hypoxia-inducible factor-2α (HIF-2α) in CRC. The function of HIF-2α is closely connected with “stem cell-like” properties, and the function of promoter upstream transcripts (PROMPTs) is often associated with the adjacent protein-coding transcripts. lncRNA-HIF2PUT expression is correlated with HIF-2α levels in CRC tissues; knockdown of lncRNA-HIF2PUT blocked HIF-2α expression, and inhibited CSC properties in CRC cell lines. Knockdown of LncRNA-HIF2PUT via siRNA decreased expression of stemness genes, impaired colony formation, decreased spheroid formation ability, retarded migration, and decreased invasiveness [48]. The well-known lncRNA HOTAIR also participates in stemness maintenance in CRC. Dou et al demonstrated CD133(+)-shHOTAIR exhibited decreased cellular proliferation, migration, invasion, colony-forming properties of in vitro CRC cells, as well as decreased Vimentin expression with increased E-cadherin expression. Down-regulation of HOTAIR expression in CD133(+) CSCs markedly decreased tumor growth and lung metastasis in xenograft nude mice [49]. Therefore, HOTAIR may be a potential therapeutic target against CRC CSCs. In addition, the lncRNA Lnc34a is abundant in the CSCs of CRC, and initiates asymmetric division by directly targeting miR-34a, disrupting spatial balance. Lnc34a recruits Dnmt3a via PHB2 and HDAC1, methylating and deacetylating the miR-34a promoter simultaneously, epigenetically silencing miR-34a expression independent of its upstream regulator, p53. Lnc34a levels increase CRC CSC self-renewal and CRC growth in xenograft models. Moreover, lnc34a is overexpressed in late-stage CRCs, causing epigenetic miR-34a silencing and CRC proliferation [50]. Wang et al recently identified lincRNA-p21 (large intergenic non-coding RNA p21) is a potent suppressor of the stem-like traits of CSCs purified from both primary CRC tissues and cell lines. LincRNA-p21 inhibited β-catenin signaling, thereby decreasing viability, self-renewal, and glycolysis of CSCs in vitro. Administration of Ad-lnc-p21-MRE significantly decreased the self-renewal potential and tumorigenicity of CSCs in nude mice [51]. In this sense, lincRNA-p21 might be promising therapeutic against CSCs in CRC.

## CLINICAL APPLICATION

LncRNAs emerge from a previously overlooked region of the genome, as a novel source of potentially useful biomarkers characterizing disease progression, recurrence, and prognosis. The fact lncRNA expression levels are typically markedly increased or decreased depending upon disease state makes them ideal for useful clinical application. Most importantly, as we discover the mechanisms of their involvement in CRC, their applicability as therapeutics against CRC may be determined (Table 6).

**Table 6:** Clinical application

LncRNA	In colorectal cancer, its expressions is:	Functions	Reference
DANCR	Upregulated	Serves as a potential prognosis predictor for CRC patients, associates with TNM stage, histologic grade, and lymph node metastasis, correlates with shorter overall survival and disease-free survival time.	[52]
FTX	Upregulated	Functions as an independent prognostic factor for CRC patients, associates with differentiation grade, lymph vascular invasion, and clinical stage, and predicts poorer overall survival.	[53]
PCAT-1	Upregulated	Serves as an independent prognostic factor for CRC outcome and indicates poorer overall survival.	[54, 55]
RP11-462C24.1	Downregulated	Serves as a prognosis indicator for CRC patients, associates with distant metastasis and poor disease-free survival.	[56]
PRNCR1	Upregulated	Proved to be a sensitive diagnostic biomarker of CRC.	[57]
NEAT1	Upregulated	Serves as a diagnostic and prognostic biomarker of overall survival in CRC, associates with tumor differentiation, invasion, metastasis and TNM stage, and correlates with shorter disease-free survival time and overall survival time.	[58, 59]
HOTTIP	Upregulated	Predicts unfavorable prognosis for CRC patients.	[37]

LncRNAs have been intensively investigated for their role in predicting CRC prognosis. Liu et al reported that lncRNA DANCR expression was significantly increased in CRC tissues compared to adjacent normal tissues. Increased lncRNA DANCR expression was negatively correlated with TNM stage, histologic grade, and lymph node metastasis. Moreover, increased lncRNA DANCR expression was associated with decreased overall and disease-free survival for CRC patients [52]. Therefore, lncRNA DANCR expression holds potential as a prognosis predictor for CRC. FTX is another lncRNA with prognostic potential in CRC. FTX was significantly overexpressed in CRC tissues. Decreased FTX expression is associated with decreased differentiation grade, lymphatic vascular invasion, and clinical stage. Further multivariate analyses revealed that increased FTX levels serves as an independent prognostic factor for CRC patient survival [53].

LncRNA PCAT-1 (prostate cancer-associated ncRNA transcripts 1) is involved with human prostate cancer progression [54]. Ge et al demonstrated markedly increased PCAT-1 expression in CRC tissues. Increased PCAT-1 levels are associated with distant metastasis, and poorer overall survival. Multivariable Cox regression analysis identified PCAT-1 overexpression is an independent prognostic factor for CRC outcome. [55].

Some lncRNAs may also hold promise in the future as novel diagnostic biomarkers for CRC patients. lncRNA RP11-462C24.1 expression is decreased in CRC cancer tissues. Additionally, the levels of RP11-462C24.1 are decreased in CRC patients with metastatic disease. Multivariate analysis identified RP11-462C24.1 to be an independent predictor of patient prognosis. Decreased RP11-462C24.1 levels were also associated with decreased disease-free survival. RP11-462C24.1 may be a novel prognostic and diagnostic marker for CRC [56]. In addition, prostate cancer non-coding RNA 1 (PRNCR1) has also been investigated as a diagnostic marker for CRC. Yang et al investigated the clinical significance and biological function of PRNCR1 in CRC. In a cohort of 63 patients, PRNCR1 was significantly overexpressed in CRC tissues compared to adjacent control tissues, and PRNCR1 may be a sensitive diagnostic biomarker of CRC [57].

Liu et al demonstrated NEAT1 (nuclear-enriched abundant transcript 1) expression was increased in colorectal cancer, and positively associated with tumor differentiation, invasion, metastasis, and TNM stage. Increased NEAT1 expression levels were correlated with decreased patient survival. More importantly, Cox's proportional hazards analysis demonstrated increased NEAT1 expression was an independent prognostic marker of poor CRC patient outcome. Therefore, NEAT1 may be a CRC prognostic indicator [58]. Wu et al analyzed the expression of NEAT1 in blood, matched primary tumor tissues, para-tumor tissues, metastatic tissues, and immune cells from CRC patients and normal controls. Whole blood NEAT1 expression was significantly increased in colorectal cancer patients. Moreover, an elevated expression of NEAT1 was also seen in neutrophils from CRC patients [59]. In this sense, whole blood NEAT1 expression may be a novel diagnostic, prognostic, and survival biomarker in colorectal cancer. Apart from that, Ren et al characterized the role of a new lncRNA HOTTIP (HOXA transcript at the distal tip) in CRC, and demonstrated HOTTIP was increased in CRC tissues, and positively correlated with clinical stage and distant metastasis. Multivariate analysis suggests HOTTIP overexpression to be an independent factor of poor CRC prognosis [37]. These lncRNAs are potentially valuable in clinical practice. However, there is still a long way for them to go from bench to bedside.

## CONCLUSION AND PERSPECTIVES

More than 1.2 million patients are diagnosed annually with colorectal cancer, one of the most common human malignancies. Comprehension of the underlying molecular pathogenesis of the disease is urgently needed. It is now recognized that mutations within the noncoding genome are major determinants of human diseases [60]. LncRNAs, which occupy a large size of the noncoding genome, serve as important signals of specific cellular states, and can be employed to identify cellular pathologies such as cancer. In this review, we have carefully culled data supporting the use of lncRNAs for diagnostic, prognostic, and potentially therapeutic use for CRC patients. Currently, there remain significant gaps in our understanding of lncRNA function in CRC. The function of many lncRNAs in CRC development and progression remain unknown. Efforts devoted to elucidating the underlying molecular mechanisms of CRC-specific lncRNAs are warranted and necessary. Systematic identification of potentially important lncRNAs central to CRC pathophysiology is necessary to advance the medical treatment of CRC for generations to come.
